# Supplementary material for: Centrosome Positioning in Migrating Dictyostelium Cells
Source: Cells. 2022 May 29;11(11):1776. doi: 10.3390/cells11111776 (PMC9179490; doi:10.3390/cells11111776)
Supplement: Supplementary file 1 [file cells-11-01776-s001.zip › cells-1702487-supplementary.pdf]

cells

MDPI

Communication

Centrosome positioning in migrating *Dictyostelium* cells

Supplementary Materials

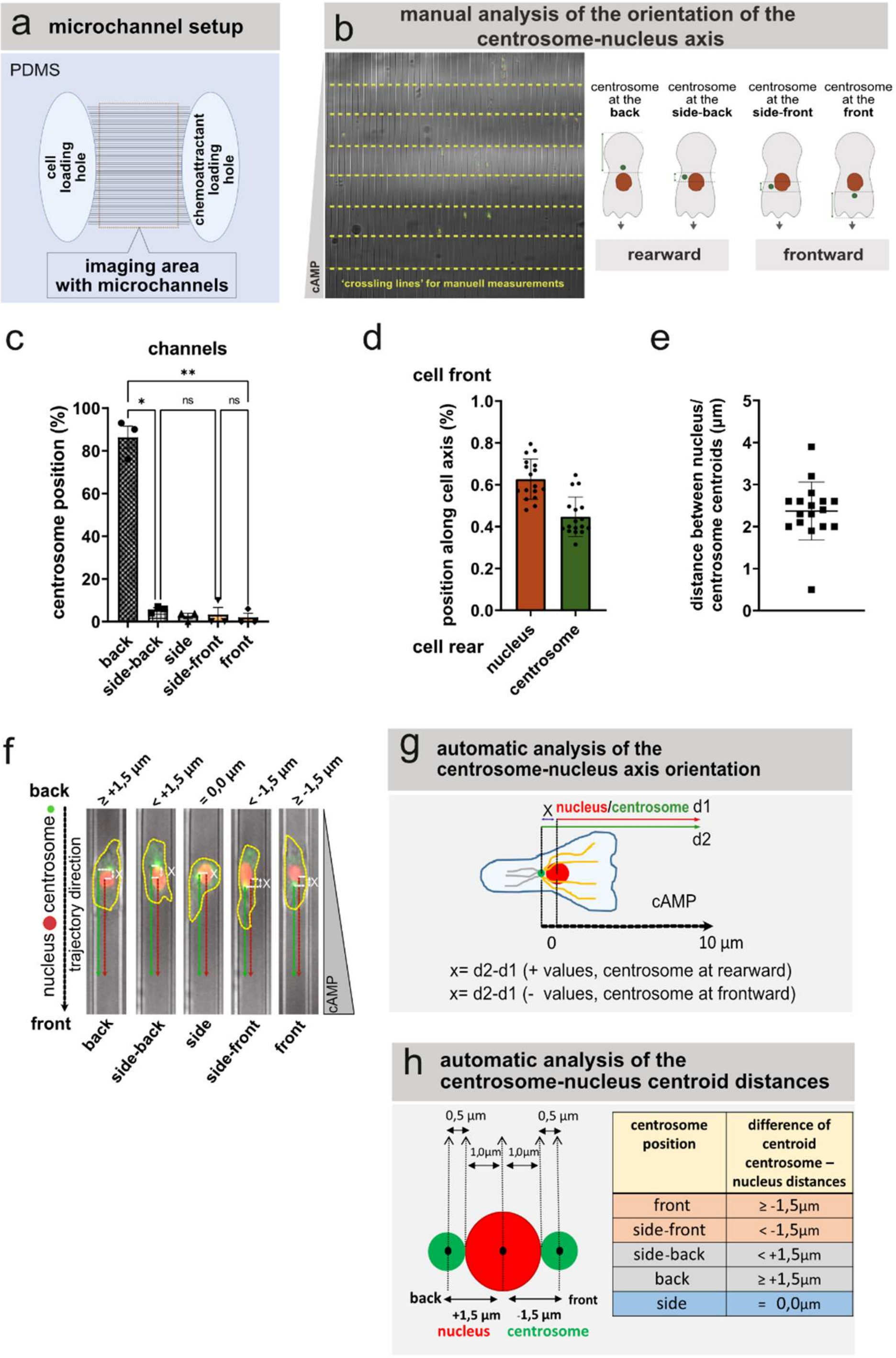

Figure S1. Microchannel setup and manual/automatic analysis of centrosome positioning. (**a**) Scheme of the PDMS device containing microchannels with one loading hole for cells and one loading hole for the chemoattractant. (**b**) Scheme illustrating the categories used for the manual quantification of the centrosome position in *Dictyostelium* cells. (c) Manual quantification of the centrosome position of *Dictyostelium* cells migrating in microchannels along a gradient of cAMP. *N* = 3, number

cells = 17. (**d**) Manual quantification of the positioning of the centrosome and the nucleus along the cell axis during migration in microchannels along a cAMP gradient (0 = cell rear; 1 = cell front). *N* = 3, number of cells = 17. (**e**) Manual quantification of the centrosome and the nucleus distances along the cell axis during migration in microchannels along a cAMP gradient. *N* = 3, number of cells = 17. (**f**) Representative microscopy images of a cell migrating in a microchannel along a gradient of cAMP showing how the centroid distances of the centrosome minus the nucleus centroid distances, were used to classify the centrosome position in relation to the nucleus. (**g**) Scheme illustrating how the automatic quantification was accomplished to classify the centrosome position relative to the nucleus. The distance of the nucleus and centrosome centroids were determined in relation to a reference point (where the cAMP or folate was released). The centroid centrosome distance (d2) minus the centroid nucleus distance (d1) were subtracted. Positive values (+) indicate centrosome is located behind, and negative values (-) indicate that the centrosome is positioned in front of the nucleus. (**h**) Table giving the threshold values used for automatic determination of centrosome-nucleus centroids as shown in (g).

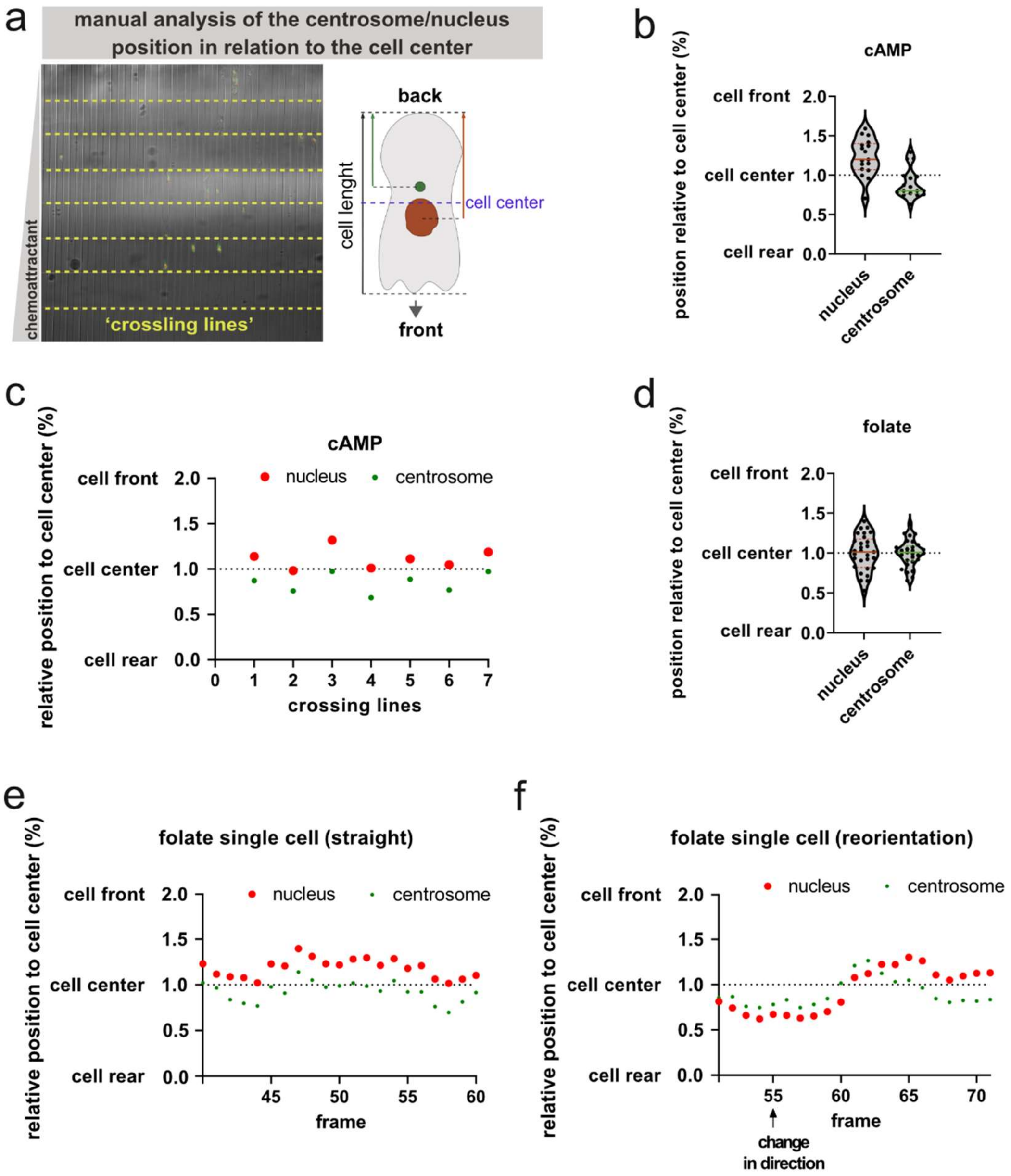

Figure S2. Position of nucleus and centrosome centroids in relation to the cell center. (**a**) Scheme illustrating the categories used for the manual quantification of the nucleus/centrosome position in relation to the cell center of *Dictyostelium* cells. Seven transverse lines were established as reference (yellow), where only the cells crossing these lines (crossing lines), were quantified for cell length,

cell center, and the distances of nucleus and centrosome centroids to the posterior edge of the cell were measured. To obtain the relative distances of the nucleus and centrosome to the cell center, the nucleus and centrosome centroid distances where divided by the distance of the cell center to the cell rear, giving the value 1.0 as the cell center. (**b** and **d**) Violin plots showing the distribution of nucleus and centrosome centroids to the cell center of *Dictyostelium* cells migrating in microchannels along a gradient of cAMP (b) or folate (d). *N* = 3, number cells = 17 for the cAMP, and *N* = 3, number cell = 29 for the folate experiments. The red and green continuous lines indicate the median, and the dashed lines (red and green) indicate the 25% and 75% quartiles. (**c**) Manual quantification of the relative position of nucleus and centrosome centroids to the cell center of a single cell migrating in microchannels along the seven reference lines in a gradient of cAMP. (**e-f**) Single-frame analysis of the nucleus and centrosome distances relative to the cell center of single cells moving in a gradient of folate. During straight migration, the nucleus is preferentially located at the cell front and the centrosome is positioned closer to the cell center (**e**). After changing direction, the nucleus re-orientates towards the front of the cell, and the centrosome is positioned behind the nucleus (**f**). The cells analyzed in (e and f) correspond to Video 7. .

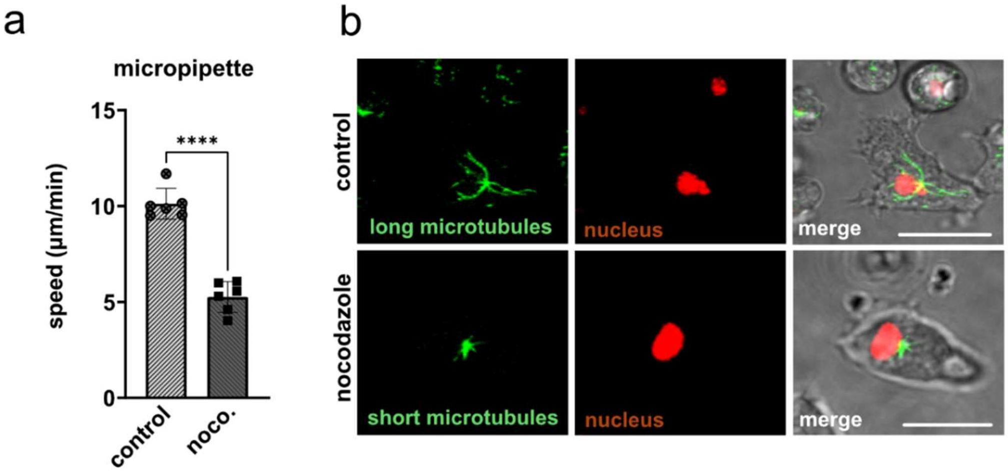

Figure S3. Nocodazole treatment results in decrease of cell speed and shorter microtubules in *Dictyostelium* cells. (**a**) Histograms shows migration speed of *Dictyostelium* cells treated with 30 µM of nocodazole for 1 h in comparison to untreated cells (control). (**b**) Confocal images of a *Dictyostelium* cell expressing GFP-tubulin (green) and mRFP-histone (red) (control), compared to a nocodazole-treated cell. After application of nocodazole, within 1 h long microtubules are massively shortened, and only short stumps emerge from the centrosome. Scale bar is 10 µm.

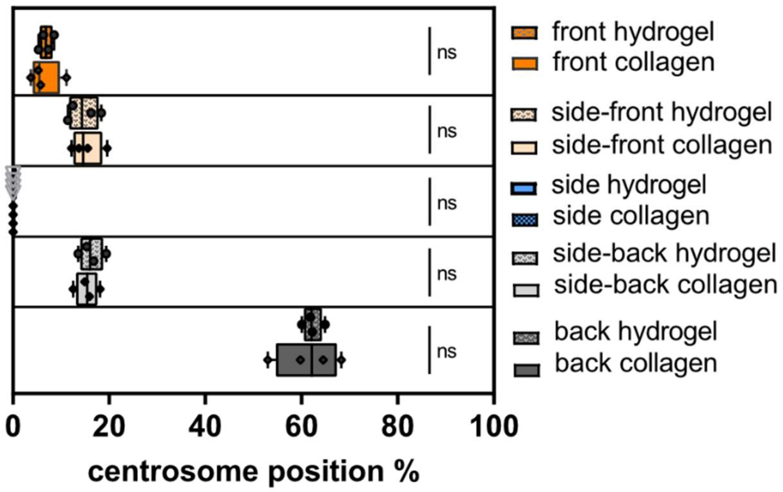

Figure S4. Positioning of the centrosome relative to the nucleus in aggregation competent *Dictyostelium* cells migrating chemotactically in 3D hydrogel or collagen type I matrices. The centrosome position shows a similar distribution under both conditions of folate chemotaxis.

Videos (available only online)

Video S1. The centrosome of *Dictyostelium* cells migrating in microchannels along a cAMP gradient preferentially locates rearward of the nucleus. *Dictyostelium* cells expressing GFP-tubulin (to visu-alize the centrosome) and mRFP-histone (nucleus) were starved for 8 to 10 h and loaded into a mi-crochannel with a cAMP gradient. Gradient orientation in video: the higher cAMP concentration is at the bottom. The time series was recorded with a frame interval of 10 s. During directed migration of cells in the narrow channels, the centrosome is predominantly found behind the nucleus. Time in min is displayed at the top left. The dots covering nucleus and centrosome in the second part of the movie were generated with the Imaris software. The green dot represents the centrosome, and the red dot is the nucleus. The tracks display the trajectory of the cell toward the chemoattractant source.

Video S2. The centrosome of *Dictyostelium* cells migrating in a micropillar array along a cAMP gra-dient preferentially locates rearward of the nucleus. *Dictyostelium* cells expressing GFP-tubulin (cen-trosome) and mRFP-histone (nucleus) were starved for 8 to 10 h and loaded into a micropillar field with a cAMP gradient. Gradient orientation in video: the higher cAMP concentration is at the bot-tom. The time series was recorded with a frame interval of 10 s. During migration of the cells through the array of micropillars, the centrosome is predominantly positioned rearward of the nu-cleus. Time in min is displayed at the top left. The dots covering nucleus and centrosome in the second part of the movie were generated with the Imaris software. The green dots represent the centrosomes, and the red dots are the nuclei. The tracks display the trajectories of the cells toward the chemoattractant source.

Video S3. The preferential position of the centrosome of *Dictyostelium* cells during migration toward cAMP released from a micropipette is rearward of the nucleus. *Dictyostelium* cells expressing GFP-tubulin (centrosome) and mRFP-histone (nucleus) starved for 8 to 10 h, are migrating along a gra-dient of cAMP released from a micropipette tip. The position of the micropipette is visible at the upper left side. The centrosome is predominantly positioned rearward of the nucleus. The time se-ries was recorded with a frame interval of 10 s. Time in minutes is displayed at the top left. The dots covering nucleus and centrosome in the second part of the movie were generated with the Imaris software. The green dot represents the centrosome, and the red dot is the nucleus. The tracks display the trajectory of the cell in the direction of the chemoattractant source.

Video S4. In *Dictyostelium* cells with disrupted microtubules, the position of the centrosome changes frequently from the back to sides and front of the nucleus during migration in a gradient of cAMP released from a micropipette. *Dictyostelium* cells expressing GFP-tubulin (centrosome) and mRFP-histone (nucleus) were starved for 8 to 10 h, and treated with 30 μM nocodazole for 1 h, are migrat-ing along a gradient of cAMP released from a micropipette tip. The position of the micropipette is visible at the upper left side. When the microtubule network is disrupted, the position of the cen-trosome relative to the nucleus is much more frequently changing compared to untreated control cells. The time series was recorded with a frame interval of 10 s. Time in minutes is displayed at the top left. The dots covering nucleus and centrosome in the second part of the movie were generated with the Imaris software. The green dot represents the centrosome, and the red dot is the nucleus. The tracks display the trajectory of the cell in the direction of the chemoattractant source.

Video S5. The centrosome of *Dictyostelium* cells is predominantly located behind the nucleus during migration in 3D environments. *Dictyostelium* cells expressing GFP-tubulin (centrosome) and mRFP-histone (nucleus) were starved for 8 to 10 h. Then, the cells were mixed with hydrogel, and the mixture was loaded immediately into the middle channel of a 3D chemotaxis chamber (ibidi). cAMP was loaded into one outer compartment of the channel and PBS into the other one. Gradient orien-tation in video: the higher cAMP concentration is at the bottom. Time series was recorded with a frame interval of 10 s. The dots covering nucleus and centrosome in the second part of the movie were generated with the Imaris software. The green dot represents the centrosome, and the red dot is the nucleus. The tracks display the trajectory of the cell in the direction of the chemoattractant source.

Video S6. In *Dictyostelium* cells moving in 3D environments, the position of the centrosome is more variable relative to the nucleus when microtubules are disrupted**.** *Dictyostelium* cells expressing GFP-tubulin (centrosome) and mRFP-histone (nucleus) starved for 8 to 10 h and treated with 30 μM nocodazole for 1 h, were mixed with hydrogel. The hydrogel-cell mix was loaded into the middle channel of a 3D chemotaxis chamber (ibidi). cAMP was loaded into one end compartment, and PBS

into the other one of the channel. Gradient orientation in video: the higher cAMP concentration is at the bottom. The cells loaded into the middle channel were recorded with a time interval of 10 s per frame. The position of the centrosome relative to the nucleus is much more variable compared to untreated control cells. The dots covering nucleus and centrosome in the second part of the movie were generated with the Imaris software. The green dot represents the centrosome, and the red dot is the nucleus. The tracks display the trajectory of the cell in the direction to the chemoattractant source.

Video S7. The centrosome of *Dictyostelium* cells migrating in microchannels along a gradient of folate preferentially locates rearward of the nucleus. Growth phase *Dictyostelium* expressing GFP-tubulin (centrosome) and mRFP-histone (nucleus) were loaded into microchannels with a folate gradient. Gradient orientation in video: the higher folate concentration is at the bottom. The time series was recorded with a frame interval of 10 s. During directed migration of cells in the narrow channels, the centrosome is predominantly found behind the nucleus. Time in minutes is displayed at the top left. The dots covering nucleus and centrosome in the second part of the movie were generated with the Imaris software. The green dots represent the centrosomes, and the red dots are the nuclei. The tracks display the trajectories of the cells toward the chemoattractant source.
